# A Flow Cytometry-Based Quantitative Drug Sensitivity Assay for All *Plasmodium falciparum* Gametocyte Stages

**DOI:** 10.1371/journal.pone.0093825

**Published:** 2014-04-15

**Authors:** Zenglei Wang, Min Liu, Xiaoying Liang, Salil Siriwat, Xiaolian Li, Xiaoguang Chen, Daniel M. Parker, Jun Miao, Liwang Cui

**Affiliations:** 1 Department of Entomology, Pennsylvania State University, University Park, Pennsylvania, United States of America; 2 Department of Parasitology, School of Public Health and Tropical Medicine, Southern Medical University, Guangzhou, Guangdong, P.R. China; Université Pierre et Marie Curie, France

## Abstract

**Background:**

Malaria elimination/eradication campaigns emphasize interruption of parasite transmission as a priority strategy. Screening for new drugs and vaccines against gametocytes is therefore urgently needed. However, current methods for sexual stage drug assays, usually performed by counting or via fluorescent markers are either laborious or restricted to a certain stage. Here we describe the use of a transgenic parasite line for assaying drug sensitivity in all gametocyte stages.

**Methods:**

A transgenic parasite line expressing green fluorescence protein (GFP) under the control of the gametocyte-specific gene *α-tubulin II* promoter was generated. This parasite line expresses GFP in all gametocyte stages. Using this transgenic line, we developed a flow cytometry-based assay to determine drug sensitivity of all gametocyte stages, and tested the gametocytocidal activities of four antimalarial drugs.

**Findings:**

This assay proved to be suitable for determining drug sensitivity of all sexual stages and can be automated. A *Z’* factor of 0.79±0.02 indicated that this assay could be further optimized for high-throughput screening. The daily sensitivity of gametocytes to three antimalarial drugs (chloroquine, dihydroartemisinin and pyronaridine) showed a drastic decrease from stage III on, whereas it remained relatively steady for primaquine.

**Conclusions:**

A drug assay was developed to use a single transgenic parasite line for determining drug susceptibility of all gametocyte stages. This assay may be further automated into a high-throughput platform for screening compound libraries against *P. falciparum* gametocytes.

## Introduction

Malaria remains a major public health menace throughout the tropics and subtropics and is responsible for nearly one million deaths annually. The past decade has witnessed increased investment in malaria control, and extensive international efforts have led to a considerable reduction of malaria incidence even in sub-Saharan Africa. With improved financial and technical supports, there are renewed interests in malaria elimination [1]. However, the current malaria control tools might not be sufficient for achieving this ambitious goal, and there are key knowledge gaps in our understanding of the tripartite interactions among malaria parasites, vectors and humans.

Of the four human malaria parasites, *Plasmodium falciparum* is the most prevalent species and causes the most severe malaria. In human red blood cells (RBCs), asexual replication of the parasite is associated with the morbidity and mortality of the disease, whereas the sexual stages, or gametocytes, are essential for continued transmission of the parasites from humans to mosquitoes [2], [3]. Consequently, control measures that target gametocytes need to be considered for the malaria elimination campaign. Interruption of malaria transmission has been recently emphasized as a priority task in the Malaria Eradication Research Agenda [4]. To date, vaccines that block parasite transmission are not in close sight. Moreover, most antimalarial drugs are ineffective in killing gametocytes; instead some even promote gametocyte formation [5], [6]. The only registered drug that is able to kill late-stage gametocytes and hypnozoites is primaquine (PMQ). The World Health Organization (WHO) recommends a single dose of PMQ to artemisinin combination therapy (ACT) for interrupting malaria transmission [7]. However, this drug has serious drawbacks, which compromise its widespread use during the malaria elimination phase [8], [9]. The root problem is hemolytic toxic effects in patients with glucose-6-phosphate dehydrogenase deficiency, which is commonly observed in endemic areas [10], [11]. Therefore, screening for new drugs and vaccines that are active against both developing and mature gametocytes is urgently needed.

Most assays for measuring *in vitro* drug susceptibility of asexual *P. falciparum* parasites rely on the detection of DNA replication, which are apparently not suitable for gametocyte stages due to the lack of DNA replication during gametocytogenesis. Gametocyte development in *P. falciparum* is a lengthy process with five morphologically distinctive stages, and it takes 10–12 days for gametocytes to reach maturity. Earlier attempts to assess gametocytocidal activities of drugs used microcopy [12], [13]. This method, however, is laborious and it is difficult to distinguish early gametocyte stages from asexual stages. Recently, new methods were developed based on the use of alamarBlue or hydroethidine as fluorescent markers of metabolic activities [6], [14], [15] and bioluminescence measurement of intracellular ATP levels [16], [17]. However, these methods are mostly developed for late gametocyte stages and the requirement for large numbers of gametocytes limits their uses for high-throughput screening (HTS) purposes. To circumvent this limitation, reporter lines with gametocyte-specific green fluorescent protein (GFP) and luciferase expression were developed, allowing for more accurate measurement of gametocytocidal activities of antimalarial drugs using flow cytometry (FCM) and chemiluminescence, respectively [18], [19]. Yet, these transgenic lines were generated using different promoters in order to obtain maximum reporter gene expression in early, middle, or late gametocyte stages. Therefore, it requires up to three transgenic lines for monitoring drug sensitivity during the entire period of gametocytogenesis. In this study, we report a robust FCM-based method for quantitative measurement of responses of *P. falciparum* gametocytes to antimalarial drugs based on the combination of a transgenic GFP-expressing line and synchronous gametocyte culture technique. This transgenic line is suitable for determining drug sensitivity of all gametocyte stages and may be fully automated and used for HTS of compound libraries against *P. falciparum* gametocytes. Using this assay, we analyzed the daily dynamics of sensitivity of gametocytes to several antimalarial drugs.

## Materials and Methods

### Ethics

RBCs were purchased from Biological Specialty Co. (Colmar, PA, USA, http://www.biospecialty.com/), and human serum was purchased from Interstate Blood Bank Inc. (Memphis, TN, USA, http://www.interstatebloodbank.com/). Since both RBCs and human serum were purchased from commercial sources with no personal information associated with the products, ethical approval from the Pennsylvania State University Institutional Review Board was exempted.

### Generation of a Stable GFP-expressing Line

**Figure 1:**
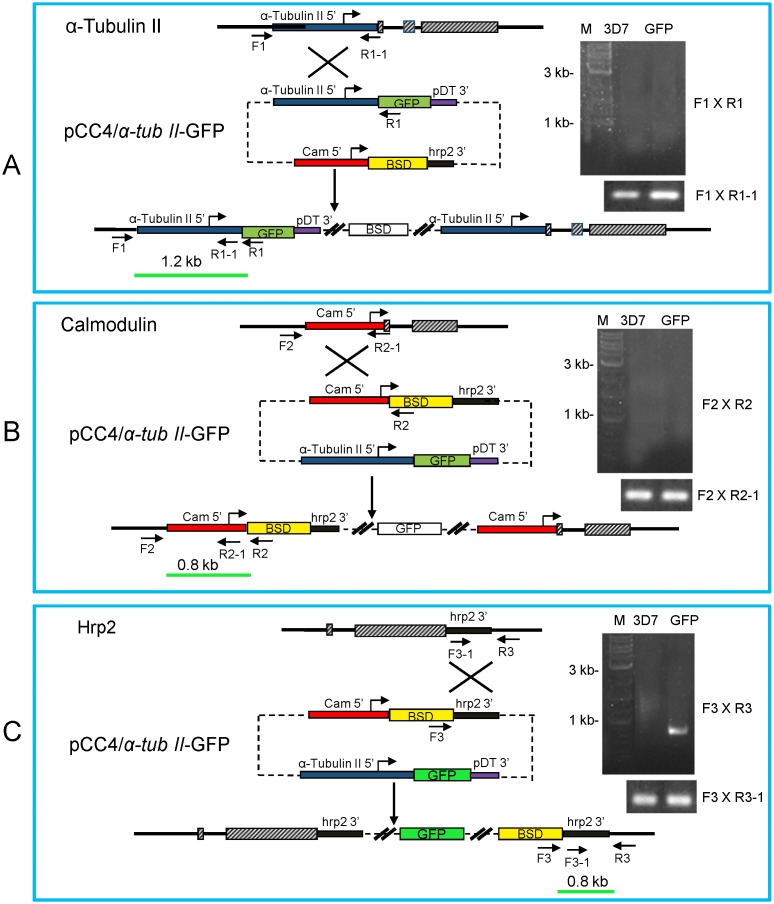
Generation of a GFP-expressing *P. falciparum* line. Schematic drawing shows the three genomic loci at *α-tubulin II*, *calmodulin*, and *hrp2* gene and the plasmid pCC4/*α-tubII*-GFP used for transfection, and three possible integration events. The plasmid contains the BSD drug selection cassette and the GFP expression cassette with the GFP expression directed by the *α-tubulin II* promoter. Shown are the predicted possible single crossover integration events into the *α-tubulin II* 5′ region (**A**), *calmodulin* 5′ region (**B**), and *hrp2* 3′ region (**C**). The positions and orientations of the primers on chromosomes and the plasmid are marked. The expected sizes of PCR products are shown as the green bars. Solid lines represent introns or intergenic regions, and hatched boxes the exons. The primer pairs F1×R1, F2×R2, and F3×R3 were used for identification of the integration events, while F1×R1–1, F2×R2–1 and F3–1×R3 were used for genomic DNA control. PCR results of the integration event are shown on the right. PCR was done with the genomic DNA from wild type (3D7) and transfected parasites (GFP). The results indicate that the integration event occurred at the *hrp2* locus (**C**).

To establish a parasite line with gametocyte-specific GFP expression, we generated a reporter cassette with the GFP open reading frame flanked by a ∼1155 bp fragment of the 5′ sequence of *α-tubulin II* gene as the promoter and the 3′ sequence of the *P. berghei* dihydrofolate reductase/thymidylate synthase (dhfr/ts) gene (pDT3′). The *α-tubulin II* promoter was amplified using primer pairs Tub 5′F and Tub 5′R (Table S1 in File S1). This reporter construct was cloned into the plasmid pCC4 at *Spe* I and *Not* I sites to replace the drug selection cassette of cytosine deaminase [20]. The resulting construct pCC4/*α-tubII*-GFP was transfected into 3D7 parasites using the method of RBC loading [21]. The transfected parasites were selected using 2.5 µg/ml of blasticidin (BSD) until parasites re-appeared in the culture. Two cycles of BSD drug on/off with 3 weeks of intervals were performed in order to enrich parasites with the plasmid integrated into the parasite genome. The integration site was determined by integration-specific PCR, with the parental line 3D7 as the control. Based on three possible scenarios of integration (Figure. 1A), three sets of primers (F1×R1, F2×R2, and F3×R3) were used to detect the integration of the plasmid at the *α-tubulin II* promoter, *calmodulin* promoter and *hrp2* 3′ region, respectively (Table S1 in File S1). Accessibility of the potential sites for homologous recombination was assessed with additional primers R1-1, R2-1 and F3-1 (Table S1 in File S1). Subsequently, parasites were cloned and the positive clones were confirmed by the visualization of GFP expression in gametocytes under a fluorescence microscope. Quantitative PCR (qPCR) was performed in order to evaluate the copy number of the GFP construct using a published method [22].

### Gametocyte Induction

*P. falciparum* parasite lines were routinely cultured in type O^+^ RBCs in complete medium supplemented with 10% human AB serum under an atmosphere of 90% N_2_/5% O_2_/5% CO_2_ [23]. Synchronous gametocytes were induced using a previously described scheme with modifications [24]. Asexual parasites were synchronized twice in two successive life cycles by 5% D-sorbitol treatment of ring-stage parasites for 10 min at 37°C. Synchronous cultures at the trophozoite stage were set up at a parasitemia of 2.5–3.2% and a hematocrit of 3% in 50 ml of complete medium in T75 flasks. On the second day (day -2), ring-stage parasitemia typically reaches to 8–12%. To induce gametocytogenesis, a part of spent medium was replaced by fresh medium. The volume of spent medium replaced depended on the parasitemia: for a parasitemia of approximately 8, 10, and 12%, 20, 25 and 30 ml of spent medium was replaced by fresh medium, respectively. On the third day (day -1), stressed schizont cultures including spent medium were transferred into T225 flasks and adjusted with fresh RBCs and medium to a parasitemia of ∼2% and a hematocrit of 3%. Cultures on day 1 were exposed to 5% D-sorbitol followed by 70% Percoll (v/v) gradient centrifugation to eliminate asexual stage parasites. From day 1 onward, 20 units/ml of heparin were added to the culture to block invasions of RBCs from residual contaminating asexual stages [25]. Medium were changed daily and Giemsa-stained smears were examined to monitor development.

### FCM

FCM analysis was performed on a Beckman Coulter XL-MCL system with 15 mW continuous laser power at 488 nm. Two band pass filters, 525 nm for the fluorescein isothiocyanate (FITC) channel and 575 nm for the phycoerythrin (PE) channel, were used to define green-emitting-only signals. Fluorescence of gametocytes was determined by documentation of green (fluorescence 1, FITC channel) and red fluorescence (fluorescence 2, PE channel). To choose a gate for quantification of green-emitting-only signals in the FITC channel, gametocytes of the transgenic GFP-expressing line 3D7*^α-tubII^* ^/GFP^ were used to define the gate. Uninfected RBCs (uRBCs), asexual-stage infected RBCs (aiRBCs) and sexual-stage parasites of parental line 3D7 were used as negative controls, which displayed no signals in the initial gate. Data were collected for 120 seconds per sample well with almost half a million events (ranging from 491,707 to 652,288). Gating counts (the events in the defined gate), and gating mean fluorescence intensity (MnX, displaying the mean fluorescence intensity of the events in the defined gate) were also collected. Gating counts were normalized by events of 600,000 using formula: normalized gating count  =  obtained gating count/event×600,000. Results were recorded as fluorescence intensity (FI) of the amount of fluorescence cells by the following formula: FI  =  normalized gating count×MnX.

### Characterization of the Assay Parameters

To compare the sensitivity of microscopy and FCM for quantifying gametocytemia, stage III gametocytes were purified by Percoll gradient centrifugation [26] to remove dead parasites, which may interfere with the assay. After purification, thin smears were made and stained by Giemsa, and 20,000 RBCs were counted by microscopy to determine gametocytemia. Then, gametocytes were diluted with RBCs to the range of 0.025–0.2%. After dilution, gametocytemia was determined in parallel by counting ∼50,000 RBCs under a microscope and counting half a million events with FCM. The results of measured gametocyte levels were plotted against the calculated values in a linear regression.

To determine whether GFP in the dying or dead cells might interfere with the assay, the ratios of signals with and without PMQ treatment were calculated using gametocytes at stages I–V. Gametocyte cultures of 3D7*^α-tubII^* ^/GFP^ at stages I–V were seeded in wells at 1% hematocrit and 0.02% gametocytemia. Eight wells were treated with a lethal dosage of 625 µM PMQ, and GFP fluorescence intensities were compared with corresponding positive control wells without drug treatment. The total volume of each well was 200 µl. After incubation for 48 h at 37°C, the cultures were applied to FCM, and the data for each pair at the same stage were collected in order to calculate the FI. The values of control wells without drug treatment were the maximum signals, and those of the drug treated wells were the minimum signals. The maximum/minimum signal ratios were calculated and analyzed. All experiments included two biological replicates each with three technical replicates. To differentiate live from dying or dead parasites, stage V gametocytes were first treated with 100 µM PMQ at 37°C for 48 h and then stained with the red fluorescent dye JC-1, a mitochondrial probe, to allow real-time visualization of live (extensive staining), dying (faint staining) or dead (no staining) gametocytes [27]. To quantify GFP and JC-1 signals by FCM, stage V gametocytes were treated with 500, 250, 125 and 62.5 µM of PMQ at 37°C for 24 h. Untreated gametocytes were used as a control. The parasites were stained with JC-1 and applied to FCM. Green fluorescence of GFP was documented by the FITC channel as described above, and afterwards red fluorescence of JC-1 was documented by the PE channel. Data were collected and shown as histograms of values of fluorescence.

### Determination of the Assay Z’ Factor

To determine the robustness of the assay, the Z’-factor statistic was determined by using uRBCs as background, and gametocytes at stage III of the PMQ treated and untreated transgenic line as negative and positive controls, respectively [28]. Negative controls were treated by 500 µM of PMQ. In a 96-well plate, uRBCs were seeded in 8 wells with a hematocrit of 1%, cultures of the negative control were seeded in 40 wells, and cultures of positive control were seeded in 48 wells. The plate was then incubated at 37°C for 48 h and analyzed by FCM. Three independent experiments were performed. The Z’ factor was calculated using the equation Z’ = 1–3 (SD_positive_+SD_negative_)/(Mean_positive_ −Mean_negative_), where SD_positive_ and SD_negative_ were the standard deviations of positive and negative controls, respectively, while Mean_positive_ and Mean_negative_ represented the mean FI values of positive and negative controls, respectively.

### *In vitro* Drug Sensitivity Assay

A final 0.02% gametocytemia was used for the drug assay in order to minimize the cost in parasite culture. To determine drug sensitivity of gametocytes, 100 µl of the cultures from day -1 to 11 were diluted in a complete medium with fresh human erythrocytes to a 2% hematocrit and 0.04% gametocytemia, and placed into each test well of 96-well plates prefilled with the test drugs to a final volume of 200 µl and final hematocrit of 1%. Because young gametocytes at day -1 (stressed schizonts) and day 0 could not be separated, gametocytemias were determined by counting with FCM the fluorescent gametocytes in culture, which contained asexual stage parasites. The plates were incubated at 37°C for 48 h. Chloroquine (CQ), PMQ and dihydroartemisinin (DHA) were purchased from Sigma (St Louis, MO, USA), while pyronaridine (PND) was obtained from Kunming Pharmaceutical Co. (Kunming, Yunnan, China). The stock solutions of CQ (100 mM), PMQ (100 mM), and PND (20 mM) were prepared in RPMI 1640, and DHA (143 mM) was dissolved in DMSO. Two-fold serial dilutions of each drug were made in a complete medium, with the concentration range of each drug shown in Table 1. For each parasite isolate and drug concentration, the assay was performed with at least three biological replicates, each with two technical replicates.

**Table 1:** The concentration ranges of tested drugs.

Days in gametocyte development	Drugs			
	Chloroquine (nM)	Primaquine (µM)	Dihydroartemisinin (nM)	Pronaridine (nM)
−1 to 0	2.44–10 000	0.03–500	0.39–400	0.61–5000
1	2.44–10 000	0.03–1000	0.49–1000	0.61–5000
2	3.05–100 000	0.07–5000	0.61–5000	0.19–50 000
3	3.05–100 000	0.07–10 000	0.61–10 000	0.19–50 000
4	3.80–500 000	0.15–20 000	0.38–200 000	0.38–100 000
5	3.80–500 000	0.15–20 000	0.38–200 000	0.38–100 000
6	3.80–1000 000	0.15–20 000	0.38–200 000	0.76–200 000
7–13	3.80–1000 000	0.15–20 000	1.9–500 000	0.76–200 000

### Data Analysis

Raw FCM data were processed using the FlowJo software. An analysis of variance (ANOVA) was done by R [29]. Half maximal inhibitory concentration (IC_50_) values of the drugs were calculated by using GraphPad Prism 5.

## Results

### Generation of Transgenic Parasites Expressing GFP in Gametocytes

**Figure 2:**
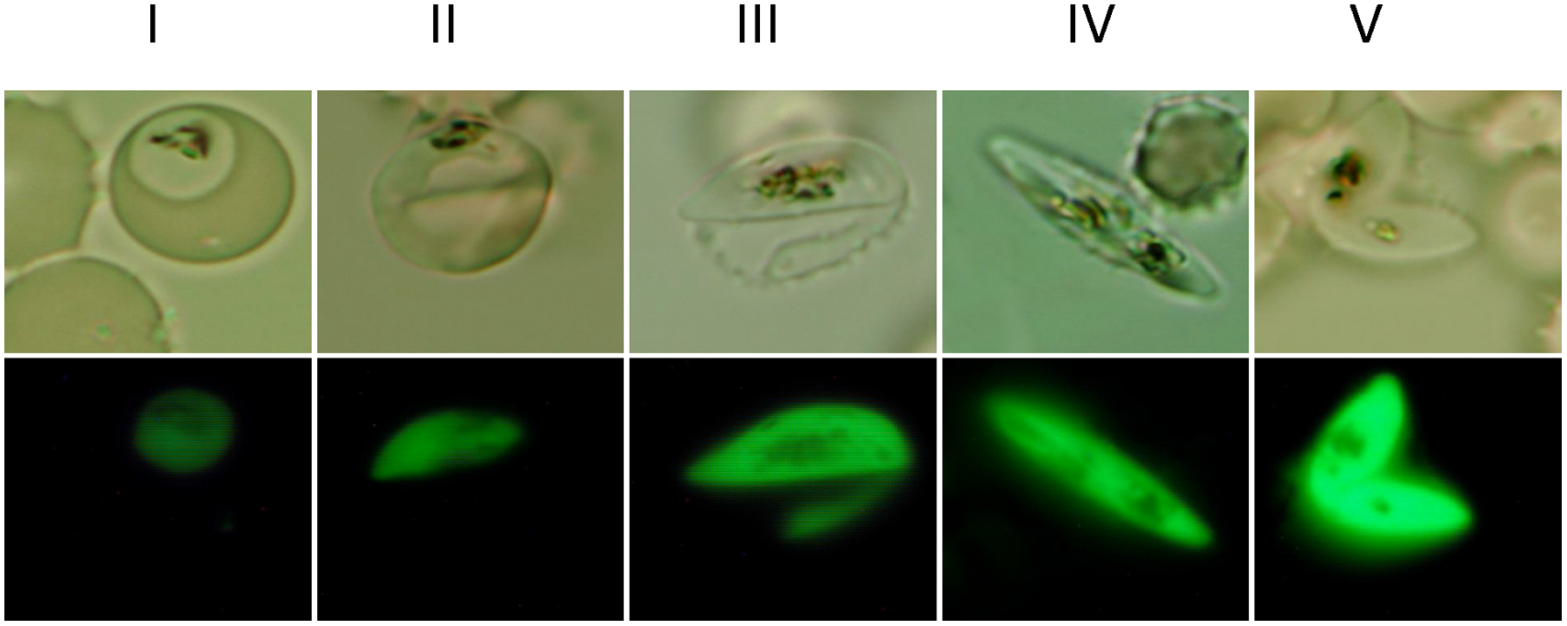
Representative images showing GFP expression in stage I–V gametocytes in the transgenic line 3D7*^α-tubII^* ^**/GFP**^ **.** Upper panel – bright field microscopic images; lower panel, GFP fluorescence.

In order to generate a transgenic parasite line with GFP expression in gametocytes, we transfected 3D7 with the pCC4/*α-tub II*-GFP construct (Figure 1). After two cycles of drug on/off selection, parasites were cloned without drug. One parasite line designated as 3D7*^α-tubII^* ^/GFP^ with strong GFP expression was selected for further characterization. This parasite line was cultured for over half a year without drug and remained GFP positive in gametocytes. Plasmid rescue from this parasite line did not yield positive clones, indicating that this parasite line contained no episomal copies of the transfected plasmid. qPCR analysis showed that there were ∼2.2 copies of the plasmid in the genome (data not shown), suggesting that the plasmid might have been integrated as a dimer. We used integration-specific PCR to determine the genomic locus of the integration. Based on the presence of three *P. falciparum* genomic fragments in the pCC4/*α-tub II*-GFP plasmid, namely, the 1 *α-tubulin II* promoter, *calmodulin* promoter, and the *hrp2* 3′ region, three sets of primers were designed to amplify the fragments covering the predicted integration sites resulted from single crossover events (Figure 1). PCR with genomic DNA of the 3D7 strain did not yield any specific PCR product, whereas PCR with the genomic DNA of the transgenic parasite line produced a 0.8 kb fragment with the primer set F3×R3, indicating that the plasmid was integrated at the *hrp2* 3′ flanking region (Figure 1C). Sequencing of the PCR product confirmed this integration event. Consistent with a previous report [30], GFP expression could be easily detected in all gametocyte stages, with GFP signal intensity increasing from stage I and reaching the highest in stage V (Figure 2). We also noticed that stressed schizont stage parasites (day -1) also expressed GFP signals, which may come from schizonts committed to gametocytogenesis, or from asexual parasites expressing the reporter [31]. The gametocyte-specific *α-tubulin II* was previously considered to be male specific [30], [32], but recently found to have promiscuous expression in both male and female gametocytes [31]. Consistently, GFP expression was observed in both sexes of gametocytes in the 3D7*^α-tubII^* ^/GFP^ line by microscopy (Figure S1 in File S1).

### Use of the Transgenic Line for Quantitation of Gametocytes by FCM

**Figure 3:**
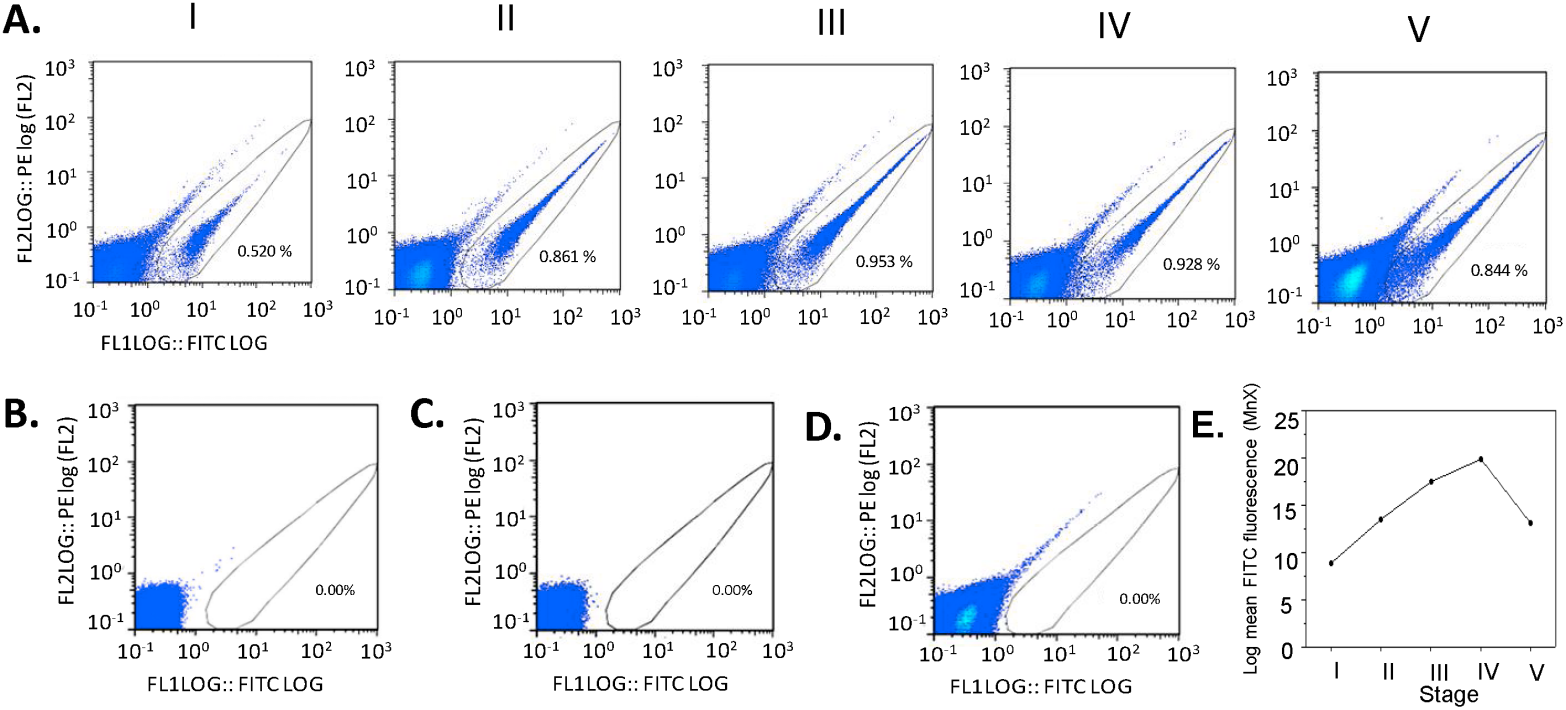
Establishment of the FCM method for GFP detection in gametocytes. (**A**) Scatter plots showing the gating signals of GFP-positive stage I–V gametocytes. Counting of gametocytes was done at relatively higher gametocytemias (%). Similar results were obtained when FCM counting of gametocytes were done at ∼0.02% gametocytemia (Figure S2 in File S1). (**B**) Uninfected RBCs (uRBC); (**C**) Asexual stages and (**D**) Gametocytes of the parental line 3D7 as negative controls. None of these controls emitted green fluorescence in the FITC channel. (**E**) Fluorescence intensity (FI) of gametocytes at different developmental stages.

For its extraordinary abilities of signal detection and potential for automation, FCM was used to determine whether the 3D7*^α-tubII^* ^/GFP^ line is suitable for quantifying gametocytes. The FITC channel (fluorescence 1) and PE channel (fluorescence 2) were used for detecting green and red fluorescence, respectively. Gating parameters were selected to specifically detect GFP fluorescence of stage I–V 3D7*^α-tubII^* ^/GFP^ gametocytes in the FITC channel (Figure 3A). In comparison, the selected gates did not detect green fluorescence in the FITC channel in uRBCs (Figure 2B), aiRBCs (Figure 2C) or 3D7 gametocyte-infected RBCs (giRBCs, Figure 2D). These cells only emitted background autofluorescence, which appeared on the diagonal line of both FITC and PE channels. For detecting GFP gametocytes, gating in the FITC channel was chosen based on the performance of these negative controls. To affirm that the selected gating specifically detects GFP-expressing gametocytes, RBCs sorted based on autofluorescence and FITC gating were examined by microscopy of Giemsa-stained smears. The results confirmed that the autofluorescent compartment only consisted of uRBCs and aiRBCs, whereas the FITC-gated part only contained gametocytes (data not shown). Under a fluorescent microscope, GFP signal appeared to increase from stage I to V (Figure 2). Using the defined FCM gating parameters, we determined the MnX of gametocytes, which increased from stage I through IV (Figure 3E). Yet, a decrease in MnX was observed in stage V gametocytes, which was reflected in the increase of cells emitting low levels of GFP fluorescence (Figure 3A). This might be due to the increase of dying and dead gametocytes in the stage V gametocyte population after being cultured for an extended period of time. This trend in GFP fluorescence during gametocyte development was confirmed in a separate experiment when FI of gametocytes was quantified under the conditions for drug assay at a 0.02% gametocytemia (Figure S2 in File S1).

**Figure 4:**
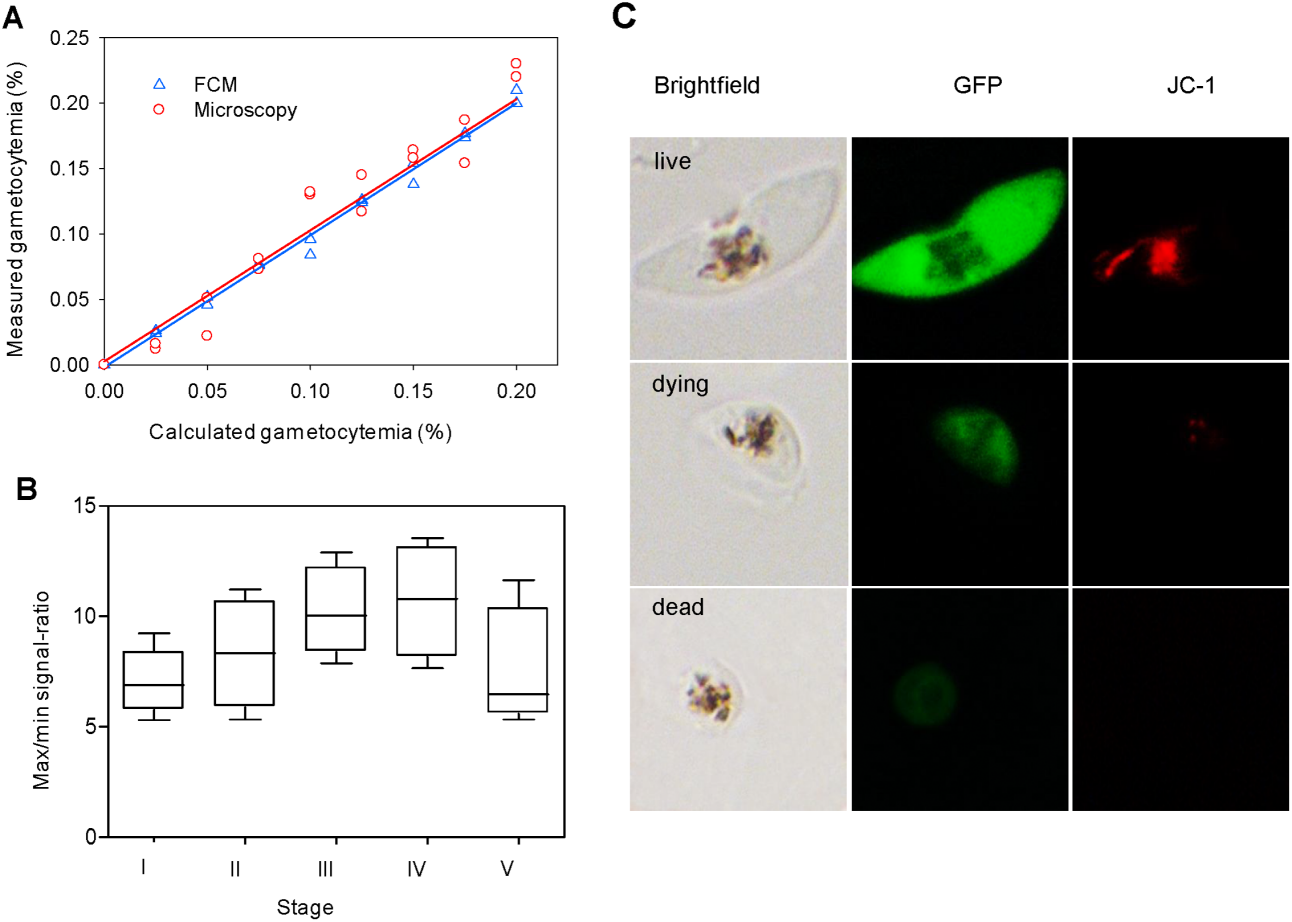
Optimization of assay parameters and characterization of gametocyte-specific GFP reporter line. (**A**) Comparison between microscopy and FCM for quantifying parasitemia. Gametocytes were serially diluted and each data point was determined in duplicates by microscopy and analyzed in parallel by FCM. The corresponding *R* ^2^ values are 0.9641 by microscopy and 0.9953 by FCM. (**B**) The maximum/minimum signal ratios at different gametocyte stages. Stage I–V gametocytes at 0.02% gametocytemia were seeded in 96-well plates and ∼half a million events were counted for each well. The box-and-whisker plot represents the ratio of fluorescence intensity (FI) for control wells to FI of these wells treated with 625 µM of PMQ. (**C**) Assessment of GFP levels in stage V gametocytes after drug treatment by fluorescence microscopy. Gametocytes emitting strong GFP fluorescence also showed strong staining of mitochondrion with JC-1, indicating of live cells (top panel). Gametocytes showing weaker GFP fluorescence also had weaker JC-1 staining of the mitochondrion, indicating dying or dead gametocytes (middle and bottom panels, respectively).

To determine whether FCM quantification provides readouts directly proportional to the number of gametocytes, we compared the FCM readout with the gametocytemia measured by microscopy. Both methods showed a linear relationship between the measured and calculated gametocytemias with an *R* ^2^ value of 0.9953 for FCM and 0.9641 by microscopy. Further, there was no significant difference between the average gametocytemias from the results of ANOVA (*F* = 0.027; *P* = 0.871), demonstrating that the FCM method for calculating gametocytemia was highly precise (Figure 4A).

Given the possibility that marker proteins such as GFP may persist for an extended period of time in dying and dead gametocytes, which may interfere with the assay, we compared the GFP fluorescence in the control and PMQ-treated gametocytes. After a lethal dose of PMQ treatment, the dying and dead gametocytes showed a >7-fold decrease in GFP fluorescence, and this change in fluorescence intensity was more pronounced in gametocyte stage III and IV (Figure 4B). Under a fluorescent microscope, treated gametocytes were smaller, with only traces of GFP fluorescence that did not completely fade away (Figure 4C). To verify that these treated gametocytes were indeed dying or dead, we stained the cells with the mitochondrial probe JC-1. The results showed that the intensities of GFP fluorescence and JC-1 staining agreed well. Live gametocytes showed both strong green fluorescence and strong red fluorescence. In contrast, parasites showing weak green fluorescence displayed weak or no red fluorescence, indicating these parasites were dead or dying. To further quantify these changes, stage V gametocytes were subjected to increasing concentrations of PMQ treatment and the GFP and JC-1 fluorescence was quantified by FCM. For the untreated control, the gametocytes emitted strong GFP and JC-1 fluorescence. With increasing PMQ concentrations, the fluorescence intensities for both GFP and JC-1 showed a similar trend of gradual decrease (Figure S3 in File S1). Parasites treated with 500 µM of PMQ (presumably dead) showed a >11-fold reduction in both GFP and JC-1 fluorescence. The contrasting FI of GFP in untreated and dying and dead gametocytes suggested that the remaining weak fluorescence should not have a significant influence on the drug assay.

### Development of an Antimalarial Drug Assay of Gametocytes

**Figure 5:**
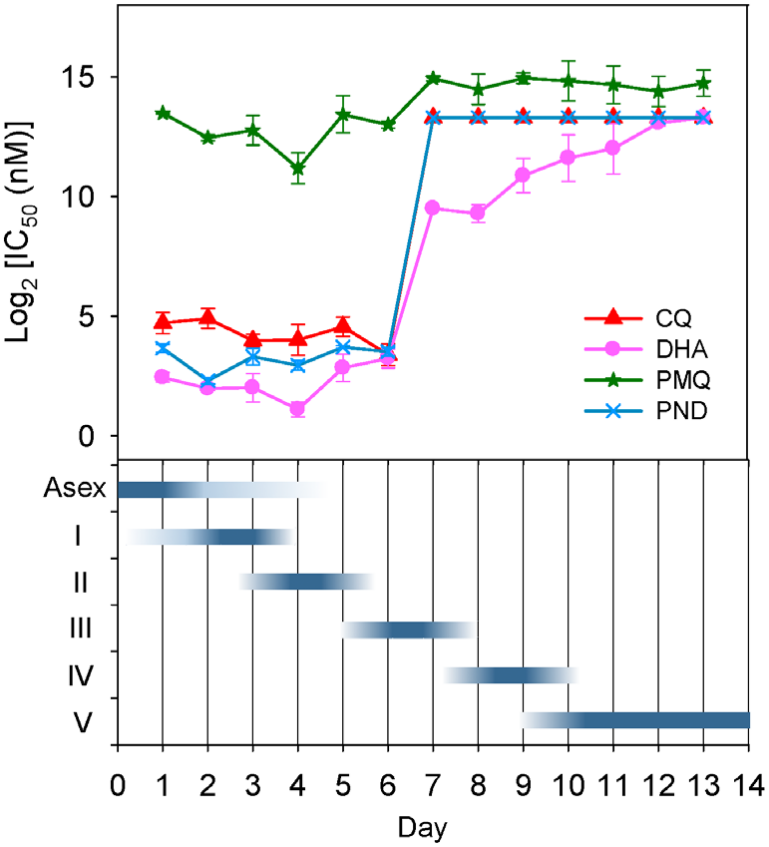
IC_50_ values of drugs on gametocytes during development. Days of gametocytogenesis are shown on the X axis. Note the drastic increase of IC_50_ values for most drugs from day 7.

The feasibility of the 3D7*^α-tubII^* ^/GFP^ transgenic line for quantification of gametocytes prompted us to further evaluate its potential for assaying drug sensitivity in gametocytes. We used four antimalarial drugs for this purpose: CQ, PMQ, DHA and PND; the latter three were reported to have gametocytocidal activities against mid- or late-stage gametocytes [15], [33], [34]. In order to assess the suitability of this transgenic line for a drug assay during the entire period of gametocytogenesis, we determined the IC_50_ values of the four drugs in gametocytes for 13 consecutive days from the stressed schizont stage (day -1 to 1) to stage V gametocyte (day 11 to 13). Gametocytes were treated with these drugs for 48 h, followed by measurement of FI by FCM. Gametocytes of the 3D7*^α-tubII^* ^/*GFP*^ reporter line were first exposed to wide ranges of drug concentrations for estimating the IC_50_s, which were then accurately measured using a narrower range of drug concentrations. As illustrated in Figure 5 and the Figure S4 in File S1, DHA, PND and CQ displayed strong inhibition activities against early gametocyte stages, with their IC_50_s being similar or slightly higher than those of asexual stages assessed by the standard SYBR Green I-based fluorescence assay. However, their gametocytocidal activities decreased sharply against stage III–V gametocytes. Specifically, sensitivity in stage III and mature gametocytes to DHA decreased by more than 70 and 1000 folds, respectively. Such a stage-specific effect was even more pronounced for CQ and PND. For PMQ, the IC_50_s of early stage (I and II) gametocytes were similar to or slightly lower than the IC_50_ of asexual stage parasites [18], which were ∼10-fold higher than the maximum plasma concentration of PMQ in the human body [35], [36]. The level of sensitivity to PMQ was only decreased by ∼3-fold from stage III and remained more or less consistent throughout the entire gametocytogenesis process.

With the verification of the transgenic line 3D7*^α-tubII^* ^/GFP^ for drug sensitivity assay in gametocytes, we further evaluated the robustness of the assay. The Z’ factor of the assay, determined from replicates in three 96-well plates, was 0.79±0.02 (values between 0.5 and 1.0 indicate an excellent assay [28], suggesting that the assay was robust and could be adapted for HTS of compounds against *P. falciparum* gametocytes.

## Discussion

Certain laboratory isolates of *P. falciparum* can readily produce gametocytes *in vitro*, among which clone 3D7 and its parental isolate NF54 are widely used as gametocyte-producer lines [37]. In this study, we generated a transgenic GFP-expressing parasite line in 3D7, 3D7*^α-tubII^* ^/*GFP*^, which was driven by the promoter of the *α-tubulin II* gene. This gene has been shown to be a male gametocyte-specific gene, which was found highly expressed in all gametocyte stages [30], [32]. However, recent research has documented its expression in both sexes of *P. falciparum* gametocytes [31], albeit the expression level was higher in male gametocytes. In the 3D7*^α-tubII^* ^/GFP^ line, robust expression of GFP was detected in all gametocyte stages, in both males and females. We adapted this reporter system to determine the drug sensitivity of all *P. falciparum* gametocyte stages.

In this assay, we used two fluorescence channels in a flow cytometer to detect GFP-positive gametocytes (FITC channel) and to rule out false-positive RBC signals (PE channel). These false positive RBCs signals normally come from autofluorescence of RBCs due to oxidative damage [38], advanced glycation end products of hemoglobin [39] and heme formation [40]. By using those two channels, the noise in our assay was almost completely removed, whereas a previous assay using a single channel for the Pfs16-GFP parasites showed high background levels of fluorescence [18]. Since the use of double channels could effectively gate the GFP-positive-only gametocytes, the actual readout from FCM was almost equal to the gametocytemia obtained by microcopy. Furthermore, we showed that the 3D7*^α-tubII^* ^/*GFP*^ parasite line provides sufficiently strong signals that could be detected by FCM from stage I to V gametocytes. A potential complication might arise if GFP protein had a long half-life and persisted in the dying and dead gametocytes, which may interfere with the drug assay [15]. Our results showed that the ratios of FI between control and PMQ-treated gametocytes were at least 7 folds, which proved sufficient for discriminating dead (and or dying) gametocytes from live ones. Another potential limitation of the parasite line is the differential expression of GFP in male and female gametocytes, which may confound data analysis if the drug is effective against only one sex of the gametocytes.

For validation of this drug assay, we found credible experimental evidence that early stages of gametocytes displayed similar drug susceptibilities to those of asexual parasites and were quite sensitive to the antimalarial drugs DHA, PND and CQ, whereas drastic decreases in sensitivity to these drugs occurred from stage III. This result is consistent with earlier findings [19], [41], [42]. In contrast, PMQ is effective in all gametocyte stages, although it also showed a slight, ∼3-fold decrease in gametocytocidal activity from stage III, implying that the mechanism of PMQ in killing gametocytes might be different from those of other drugs. Thus, stage III is an important turning point of drug susceptibility during gametocytogenesis. Coincidentally, hemoglobin digestion ceases possibly between stage III and IV gametocytes [43]. It was also observed that in late gametocyte stages there was decreased expression of genes involved in glycolysis and protein biosynthesis [44], indicating reduced metabolic activities in mature stages. Meanwhile, stage III–V female gametocytes also showed structural changes in mitochrondrion with numerous tubular cristae [42], [45]–[47], which is in sharp contrast to no or few cristae in both asexual parasites and early-stage gametocytes. While it is considered that the gametocytocidal activities of CQ and DHA for early gametocytes are due to their inhibition of hemoglobin digestion [13], [37], [48]–[50], and the similarity in structure between PND and CQ suggests that PND may also target the same pathway [19]. The molecular target of PMQ, however, seems unique and specific to gametocytes. Though its mode of action is still unknown, there is evidence that it might target mitochondrial function [37], [51]. Therefore, a better understanding of the molecular mechanisms underlying decreased sensitivity to antimalarials during the transition to stage III gametocytes is needed to develop future gametocytocidal drugs.

There is an urgent need for the development of additional antimalarial drugs with gametocytocidal activities, especially against late stage gametocytes. Several assays based on the GFP or luciferase marker [18], [19] or metabolic activities (ATP or oxidoreduction) [15], [17] have been developed and could be applicable in HTS of compounds against gametocyte stages. Our FCM-based drug assay has proved that a single transgenic parasite line may be used for assaying drug susceptibility in all gametocyte stages. This system allows for precise determination of both drug potency and kinetics of inhibition, albeit a BSD cassette in the transgenic parasite line may affect the sensitivity of compounds with similar structures. In addition, a gametocyte induction scheme with the use of heparin to eliminate asexual stages allows us to procure synchronized gametocytes at relatively high numbers [25], which are needed for adapting this assay for the HTS purpose. Furthermore, this assay, with a high Z’ factor of 0.79, is very robust, and has the potential for HTS. The capability of full automation of FCM further highlights the potential of this assay as an excellent platform for HTS. For example, to screen a library of 1000 compounds in duplicates using a setting of 0.02% gametocytemia, 1% hematocrit and 200 µl of final volume/well, it requires a total of 8 million gametocytes. A 50 ml gametocyte culture in one T75 flask would be sufficient for this even if a gametocytemia as low as 0.1% is achieved. The FCM screening process will take ∼33 hours. In addition, the time can be significantly reduced by increasing the seeded gametocytemia and decreasing the counting events. Future optimization of the conditions is needed for adaptation of this assay for HTS studies.

## Supporting Information

File S1 **Supporting table and figures. Table S1**, Primers used in this study. **Figure S1**, Mature gametocytes of the transgenic line 3D7*^α-tubII^* ^/GFP^. The graphs show strong GFP expression in both male and female gametocytes. Upper panel, GFP fluorescence. Lower panel: Bright field image showing a male gametocyte (left) with dispersed chromatin and a female gametocyte (right) with condensed chromatin. **Figure S2**, Scatter plots showing the gating signals and fluorescence intensity at 0.02% gametocytemia of stage I to V gametocytes. **Figure S3**, Comparative quantitation of GFP and JC-1 signal intensity after treatment with PMQ. The areas under curves in the histogram show the fluorescence intensities (FI) of GFP (A) and JC-1 (B) of stage V gametocytes with or without PMQ treatment. PMQ treatment was done at 62.5–500 µM. **Figure S4**, Drug response curves of stage I–V gametocytes to chloroquine (CQ), dihydroartemisinin (DHA), primaquine (PMQ) and pyronaridine (PND). The graphs show the fluorescence intensity (FI) values of the gametocytes with the error bars representing the standard error of the FI from three biological replicates.
(PPTX)
