# Supplementary material for: A Flow Cytometry-Based Quantitative Drug Sensitivity Assay for All Plasmodium falciparum Gametocyte Stages
Source: PLoS One. 2014 Apr 15;9(4):e93825. doi: 10.1371/journal.pone.0093825 (PMC3988044; doi:10.1371/journal.pone.0093825)
Supplement: File S1 — Supporting table and figures. Table S1, Primers used in this study. Figure S1, Mature gametocytes of the transgenic line 3D7α-tubII /GFP. The graphs show strong GFP expression in both male and female gametocytes. Upper panel, GFP fluorescence. Lower panel: Bright field image showing a male gametocyte (left) with dispersed chromatin and a female gametocyte (right) with condensed chromatin. Figure S2, Scatter plots showing the gating signals and fluorescence intensity at 0.02% gametocytemia of stage I to V gametocytes. Figure S3, Comparative quantitation of GFP and JC-1 signal intensity after treatment with PMQ. The areas under curves in the histogram show the fluorescence intensities (FI) of GFP (A) and JC-1 (B) of stage V gametocytes with or without PMQ treatment. PMQ treatment was done at 62.5–500 µM. Figure S4, Drug response curves of stage I–V gametocytes to chloroquine (CQ), dihydroartemisinin (DHA), primaquine (PMQ) and pyronaridine (PND). The graphs show the fluorescence intensity (FI) values of the gametocytes with the error bars representing the standard error of the FI from three biological replicates. (PPTX) [file pone.0093825.s001.pptx]

## Slide 1
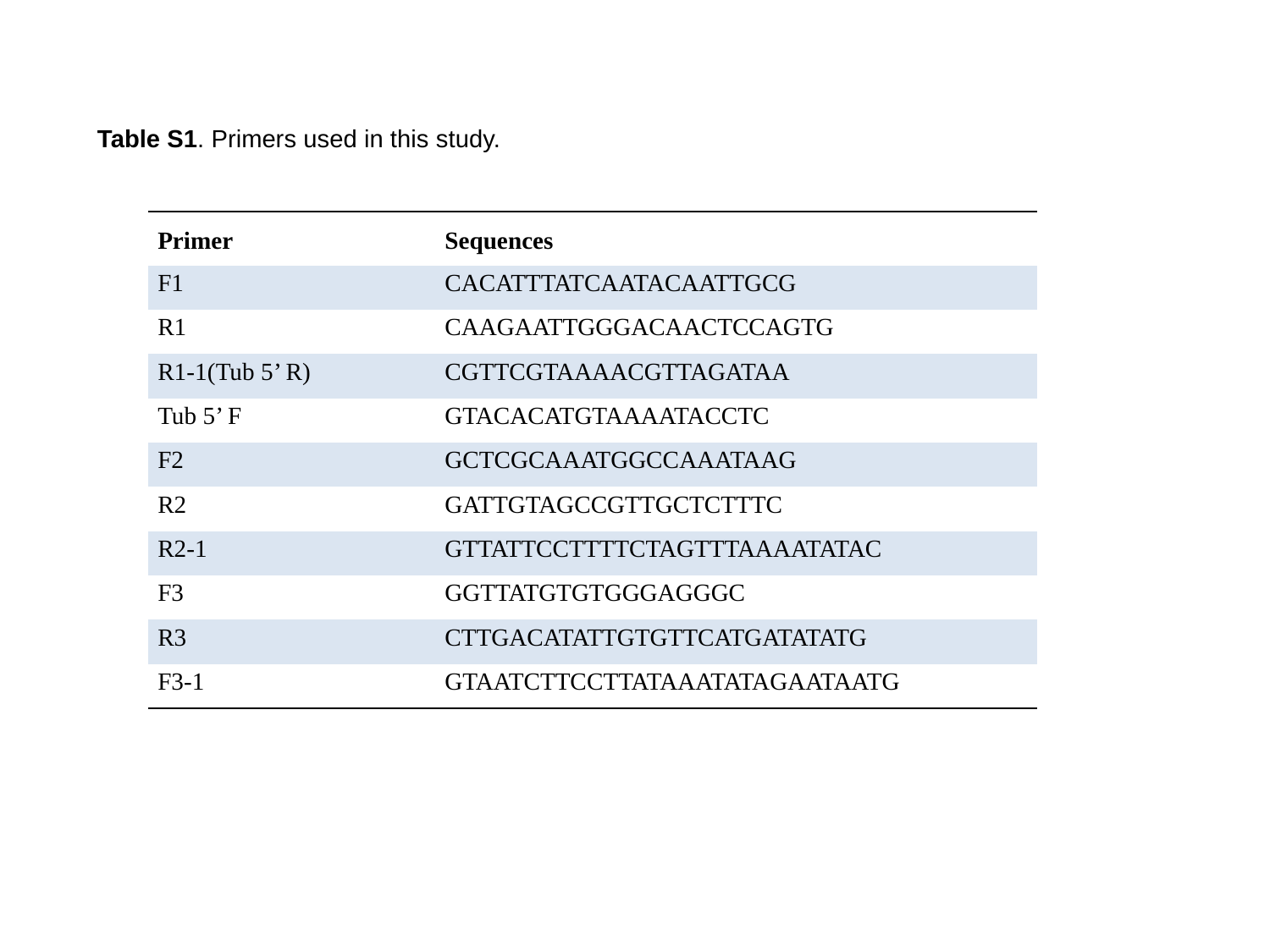

Table S1. Primers used in this study.
| Primer | Sequences |
| --- | --- |
| F1 | CACATTTATCAATACAATTGCG |
| R1 | CAAGAATTGGGACAACTCCAGTG |
| R1-1(Tub 5’ R) | CGTTCGTAAAACGTTAGATAA |
| Tub 5’ F | GTACACATGTAAAATACCTC |
| F2 | GCTCGCAAATGGCCAAATAAG |
| R2 | GATTGTAGCCGTTGCTCTTTC |
| R2-1 | GTTATTCCTTTTCTAGTTTAAAATATAC |
| F3 | GGTTATGTGTGGGAGGGC |
| R3 | CTTGACATATTGTGTTCATGATATATG |
| F3-1 | GTAATCTTCCTTATAAATATAGAATAATG |

## Slide 2
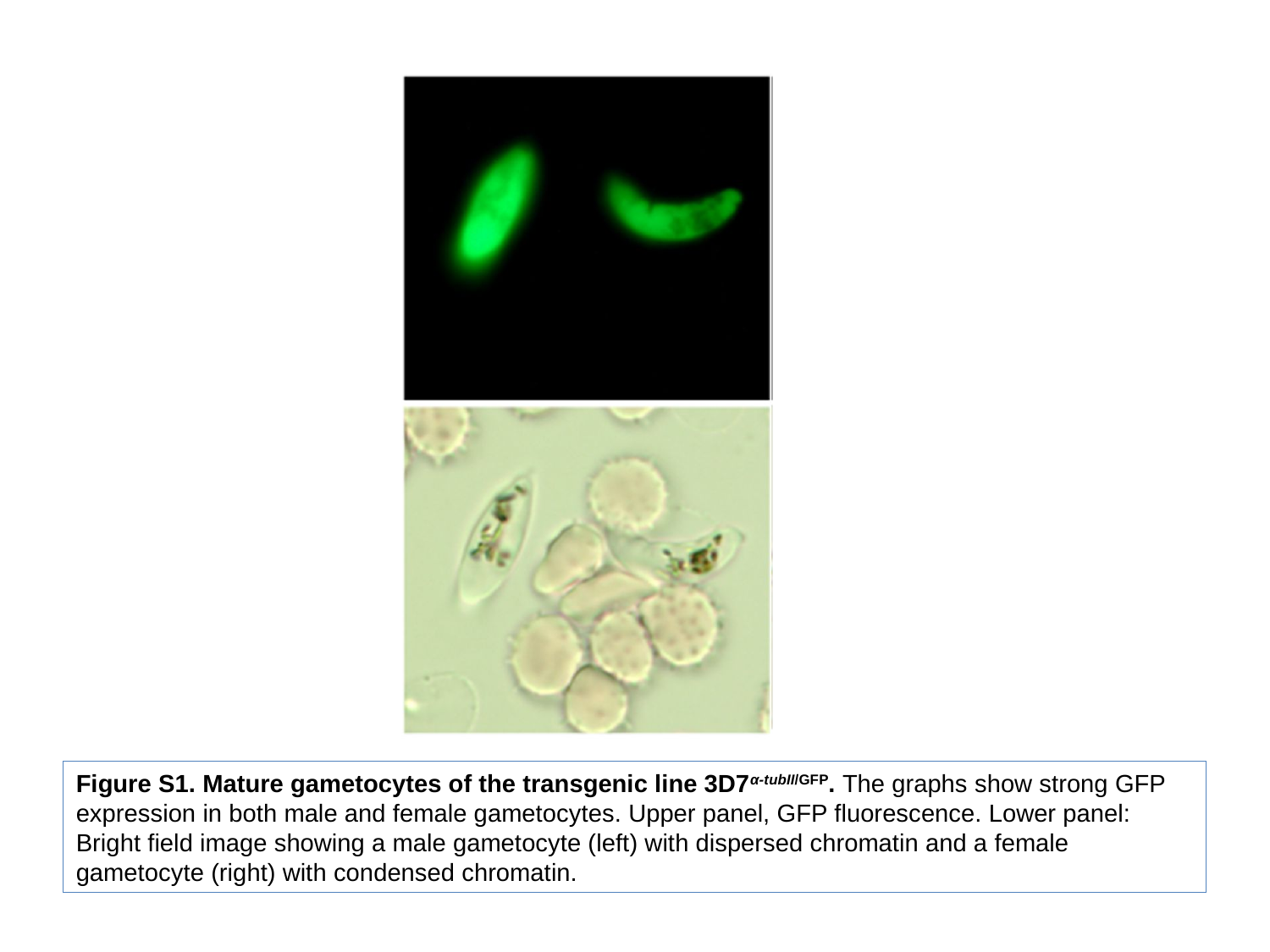

Figure S1. Mature gametocytes of the transgenic line 3D7α-tubII/GFP. The graphs show strong GFP expression in both male and female gametocytes. Upper panel, GFP fluorescence. Lower panel: Bright field image showing a male gametocyte (left) with dispersed chromatin and a female gametocyte (right) with condensed chromatin.

## Slide 3
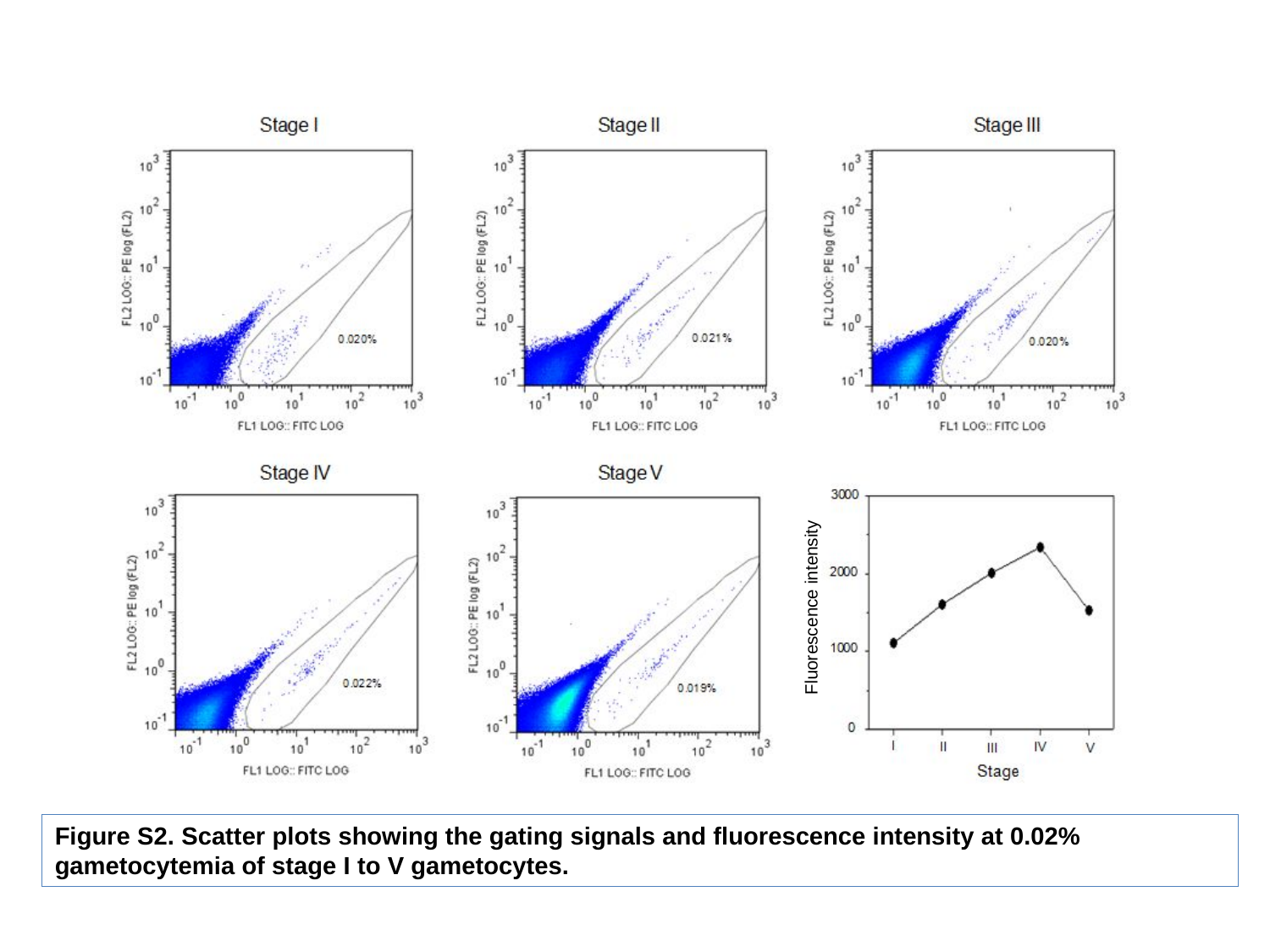

Fluorescence intensity
Figure S2. Scatter plots showing the gating signals and fluorescence intensity at 0.02% gametocytemia of stage I to V gametocytes.

## Slide 4
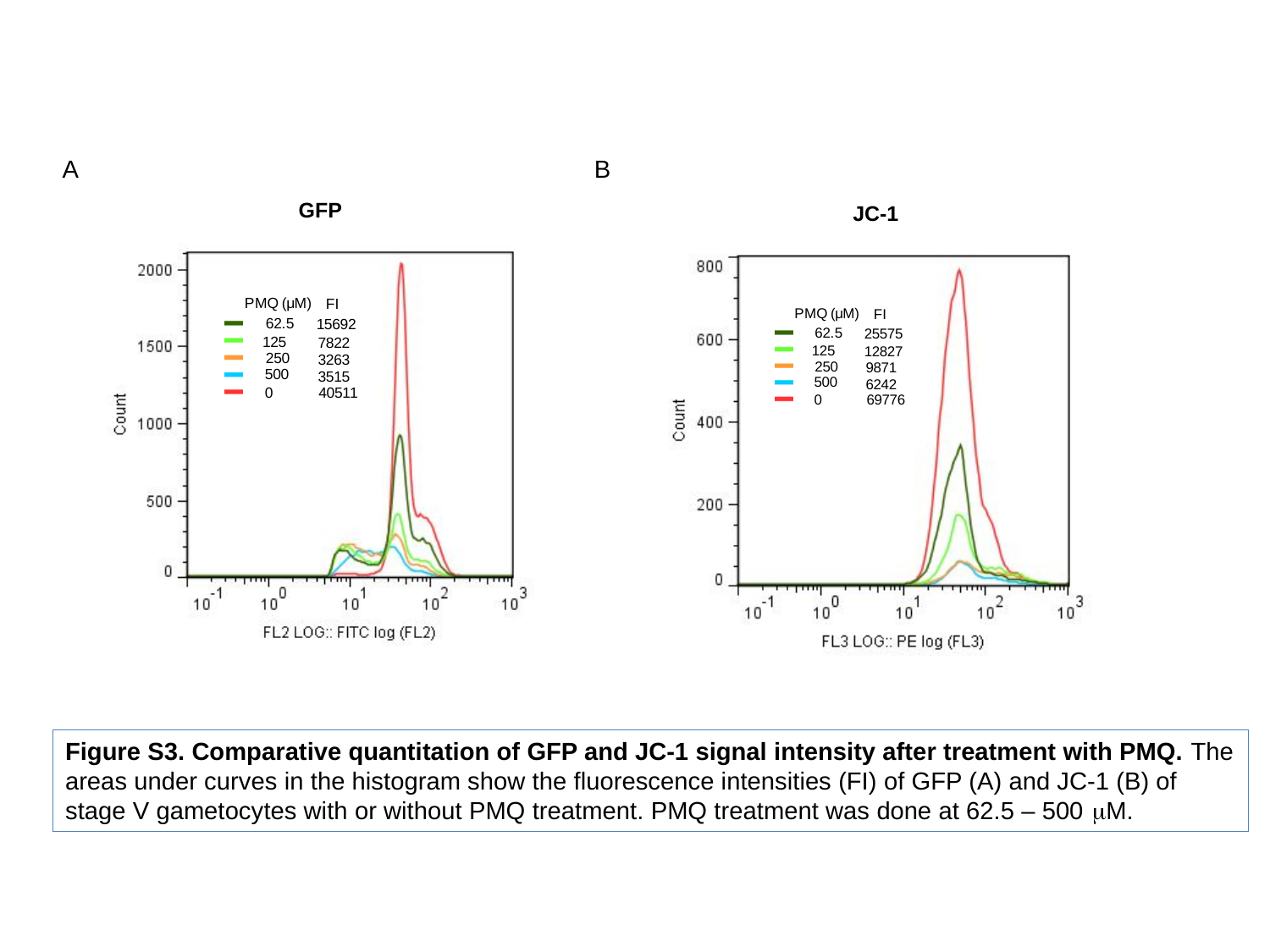

B
A
GFP
JC-1
Figure S3. Comparative quantitation of GFP and JC-1 signal intensity after treatment with PMQ. The areas under curves in the histogram show the fluorescence intensities (FI) of GFP (A) and JC-1 (B) of stage V gametocytes with or without PMQ treatment. PMQ treatment was done at 62.5 – 500 μM.

## Slide 5
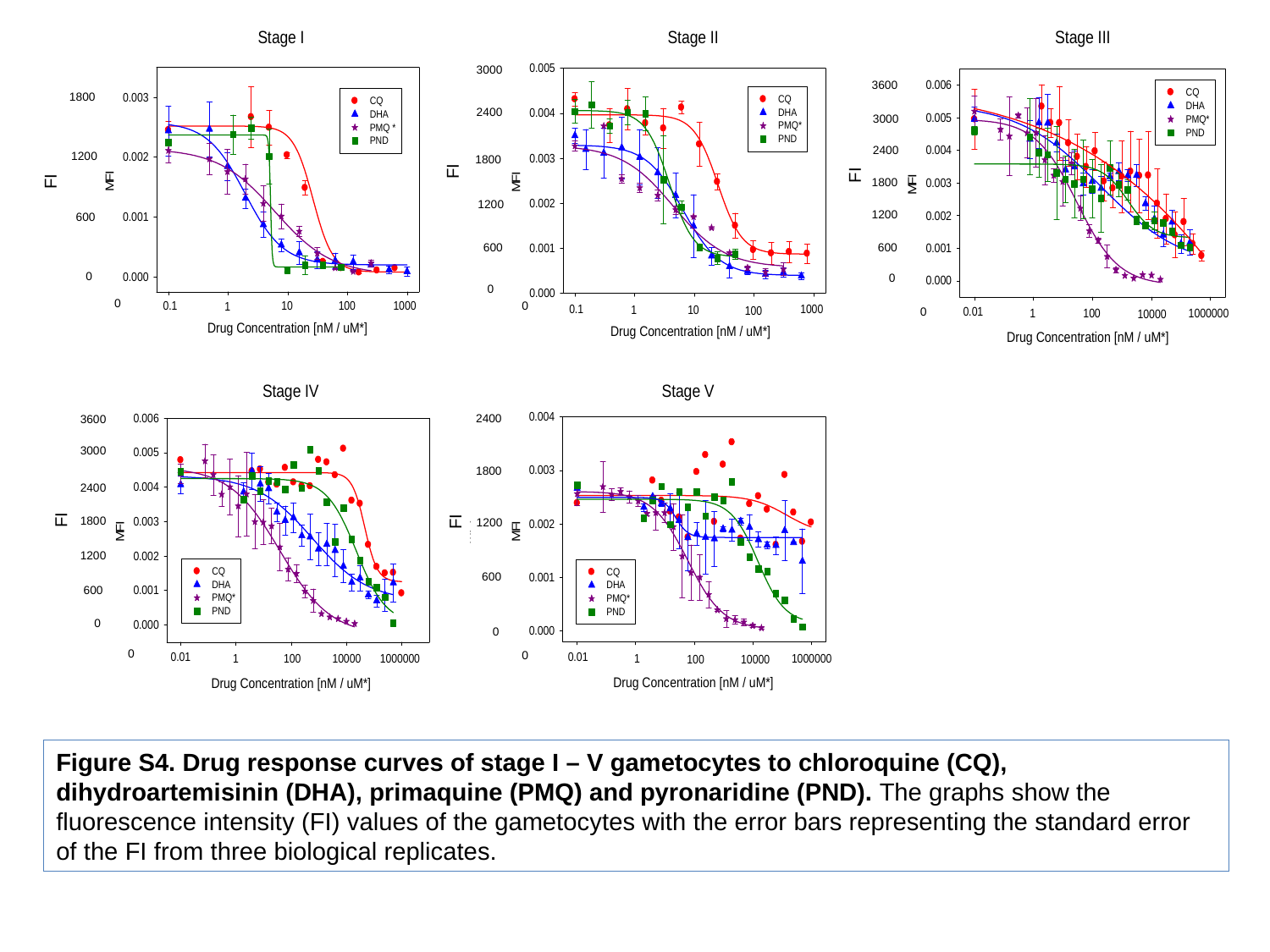

Figure S4. Drug response curves of stage I – V gametocytes to chloroquine (CQ), dihydroartemisinin (DHA), primaquine (PMQ) and pyronaridine (PND). The graphs show the fluorescence intensity (FI) values of the gametocytes with the error bars representing the standard error of the FI from three biological replicates.
